# Thirty Years with ERH: An mRNA Splicing and Mitosis Factor Only or Rather a Novel Genome Integrity Protector?

**DOI:** 10.3390/cells12202449

**Published:** 2023-10-13

**Authors:** Piotr Kozlowski

**Affiliations:** Department of Molecular Biology, Institute of Biochemistry, Faculty of Biology, University of Warsaw, Miecznikowa 1, 02-096 Warsaw, Poland; pkozlowski@biol.uw.edu.pl

**Keywords:** heterochromatin, DNA damage response, replication stress, genome integrity, piRNA, miRNA, ncRNA, *Schizosaccharomyces pombe*, *Caenorhabdtitis elegans*, *Homo sapiens*

## Abstract

ERH is a 100 to about 110 aa nuclear protein with unique primary and three-dimensional structures that are very conserved from simple eukaryotes to humans, albeit some species have lost its gene, with most higher fungi being a noteworthy example. Initially, studies on *Drosophila melanogaster* implied its function in pyrimidine metabolism. Subsequently, research on *Xenopus laevis* suggested that it acts as a transcriptional repressor. Finally, studies in humans pointed to a role in pre-mRNA splicing and in mitosis but further research, also in *Caenorhabditis elegans* and *Schizosaccharomyces pombe,* demonstrated its much broader activity, namely involvement in the biogenesis of mRNA, and miRNA, piRNA and some other ncRNAs, and in repressive heterochromatin formation. ERH interacts with numerous, mostly taxon-specific proteins, like Mmi1 and Mei2 in *S. pombe*, PID-3/PICS-1, TOST-1 and PID-1 in *C. elegans*, and DGCR8, CIZ1, PDIP46/SKAR and SAFB1/2 in humans. There are, however, some common themes in this wide range of processes and partners, such as: (a) ERH homodimerizes to form a scaffold for several complexes involved in the metabolism of nucleic acids, (b) all these RNAs are RNA polymerase II transcripts, (c) pre-mRNAs, whose splicing depends on ERH, are enriched in transcripts of DNA damage response and DNA metabolism genes, and (d) heterochromatin is formed to silence unwanted transcription, e.g., from repetitive elements. Thus, it seems that ERH has been adopted for various pathways that serve to maintain genome integrity.

## 1. Introduction

Next year will mark the 30th anniversary of the discovery of the *ERH* gene which was first reported in the fruit fly *Drosophila melanogaster* by Tsubota and coworkers in 1994 [1]. In fruit flies, hypo-morphic mutations in the *rudimentary* (*r*) gene encoding the multi-enzymatic protein that catalyzes the first three steps of the de novo pathway for pyrimidine nucleotide (UMP) biosynthesis lead to a deficiency in pyrimidines [2]. The deficiency manifests in a characteristic truncation of the wings [1]. Searching for new mutations that alter this phenotype, a change in an unknown gene that augmented it was identified. The mutation alone (i.e., in the wild-type *r* background) had no effect on the wing formation, therefore the new gene was named *enhancer of rudimentary* (*e(r)*) [1]. Its homolog from another species of the same genus, *D. virilis,* was also cloned and protein products of these two genes displayed a remarkably high degree of sequence conservation [1]. However, for the sake of accuracy, the first indication of the existence of the protein encoded by *ERH* appeared one year earlier, i.e., in 1993, when, as it later turned out, its C-terminal fragment (sequenced by automatic Edman degradation) was described as the antibacterial peptide 3910 from pig intestine [3].

## 2. Occurrence of the Gene

The first homolog of *e(r)* outside the *Drosophila* genus was found in humans and it was termed *ERH* [4]. Later, *ERH* as the acronym from *enhancer of rudimentary homolog* (Drosophila) became the official symbol of the gene. However, the current official full name of this gene according to the HUGO Gene Nomenclature Committee (HGNC ID: 3447) is “ERH mRNA splicing and mitosis factor”. Subsequently, Tsubota and coworkers identified the *ERH* homologs (coding for the proteins then called ER) in the nematode *Caenorhabditis elegans*, mosquito *Aedes aegypti*, zebrafish *Danio* (*Brachydanio*) *rerio*, mouse *Mus musculus*, and thale cress *Arabidopsis thaliana* [5].

**Figure 1:**
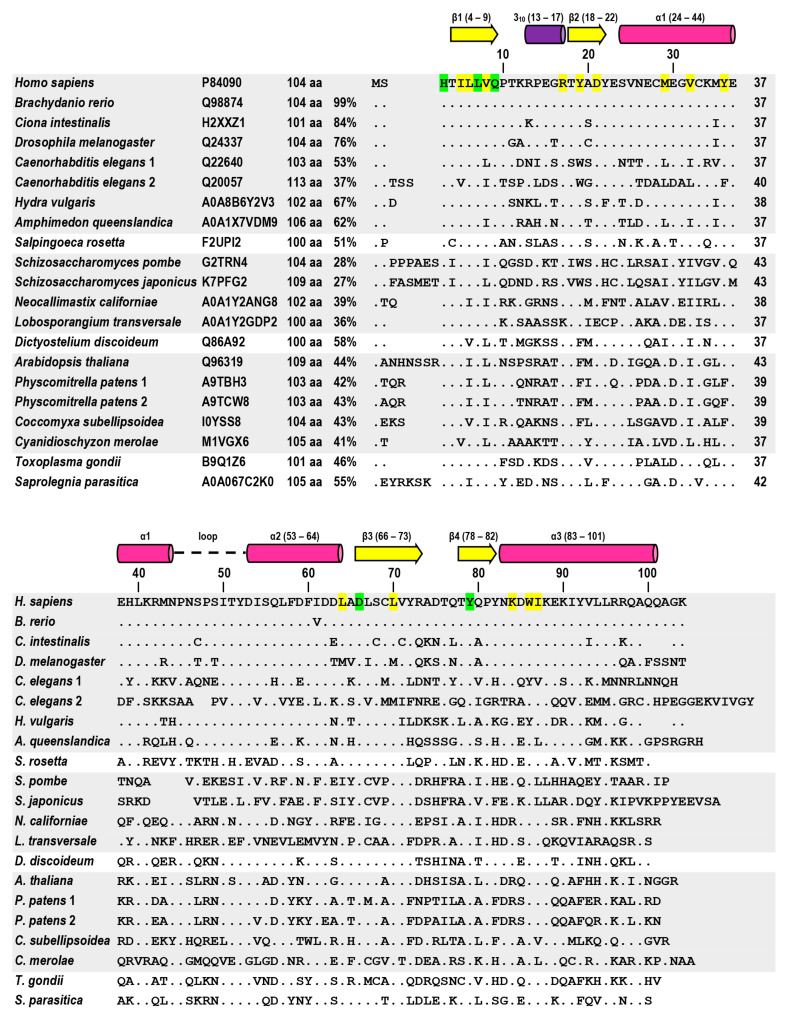
Comparison of amino acid sequences of the ERH proteins from the indicated organisms. For *C. elegans* and *P. patens,* both of their ERH paralogs are shown; the other species possess only one ERH. The species belonging to the same taxon (Metazoa, Choanoflagellata, Fungi, Amoebozoa, Archaeplastida and SAR [9]) are grouped together using the same color background. For each protein, the UniProt (www.uniprot.org) identifier, its length and its homology to the human ERH are shown. Vertical bars with numbers above the alignment indicate every tenth amino acid residue in the human ERH polypeptide chain. Numbers at the end of each line in the upper section of the alignment indicate the position of the last amino acid residue from each sequence shown in this section. A space sign signifies no amino acid residue and a dot sign indicates a residue identical with that in the human ERH. Residues in the human ERH that are identical in all sequences are highlighted in green, while those that are identical or conserved substitutions in the other sequences are highlighted in yellow. The secondary structures of the human ERH (PDB ID: 2NML) are shown above the alignment (the 3 α-helices as pink barrels, the 3_10_-helix as a violet barrel and the 4 β-strands as yellow arrows). The α-helices are designated as α1, α2 and α3 and the β-strands as β1, β2, β3 and β4 according to the order of their appearance in the polypeptide chain starting from the N-terminus; numbers in parentheses indicate the position of the structure in the chain. A dashed line denotes the α1-α2 loop.

Progress with genome sequencing (data available from NCBI and UniProt) revealed that the *ERH* gene is also present outside Bilateria, in sponges (e.g., *Amphimedon queenslandica*) and cnidarians (e.g., *Hydra vulgaris*), and in lower plants (e.g., the moss *Physcomitrium* (*Physcomitrella*) *patens*) and also in green algae (e.g., *Coccomyxa subellipsoidea*) and red algae (e.g., *Cyanidioschyzon merolae*); thus, it became obvious that the gene is common in animals (Metazoa) and plants sensu lato (Archaeplastida) (Figure 1). The gene was also found in choanoflagellates (e.g., *Salpingoeca rosetta*). However, in fungi belonging together with animals and choanoflagellates to opisthokonts, it was identified in lower ones, e.g., in Chytridiomycota (e.g., *Neocallimastix californiae*) or Mucoromycota (e.g., *Lobosporangium transversale*), but not in Basidiomycota (e.g., the king bolete *Boletus edulis* and corn smut fungus *Ustilago maydis*) and Ascomycota (e.g., the budding yeast *Saccharomyces cerevisiae* and filamentous fungus *Aspergillus nidulans*), with the single exception within the latter, i.e., the fission yeasts of the *Schizosaccharomyces* genus [6,7]. Similarly, in protists it was found in some (e.g., the slime mold *Dictyostelium discoideum* belonging to the amoebozoans, and the parasites *Toxoplasma gondii* belonging to the alveolates and *Saprolegnia parasitica* belonging to the stramenopiles), while it seems to be absent from other species (e.g., the ciliate *Paramecium caudatum* and parasite *Entamoeba histolytica*). It has not been found in bacteria and archeons. The above pattern suggests that the *ERH* gene could be present in the genome of the last eukaryotic common ancestor and during evolution it was lost in some lineages, e.g., in most higher fungi. When present, in most genomes *ERH* exists as a single copy gene. There are, however, species that possess two *ERH* genes, e.g., *P. patens* and *C. elegans*.

In the human genome, there is only one *ERH* gene, which is located on chromosome 14 (location 14q24.1, Ensembl ID: ENSG00000100632). It spans 18,172 bp, and has three long introns and four very short exons, which encompass a CDS of 315 bp that codes for the 104-aa protein (including the initial methionine) with a theoretical molecular weight of 12,259 Da. There are also two pseudogenes. One of them, ERH pseudogene 1 (*ERHP1*, HGNC ID: 41916), is located on chromosome 7 and it is this location (7q34) that *ERH* was mistakenly initially assigned to by fluorescence in situ hybridization (FISH) [4]. The other, ERH pseudogene 2 (*ERHP2*, HGNC ID: 41917), is located on chromosome 6 (location 6p12.1).

## 3. Organization and Regulation of the Gene

**Figure 2:**
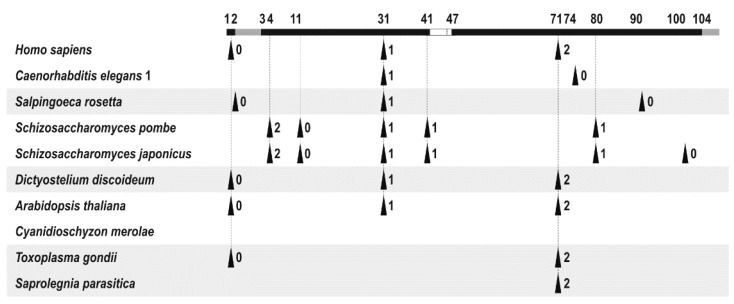
Comparison of intron positions in the *ERH* genes from the indicated organisms. The species belonging to the same taxon (Metazoa, Choanoflagellata, Fungi, Amoebozoa, Archaeplastida and SAR [9]) are grouped together using the same color background. Introns are marked with triangles and numbers that indicate their phases (0, 1 and 2) and their positions are shown above a schematic based on the human *ERH* coding sequence (CDS), represented by a black solid line with a white segment to indicate the location of the 4- and 5-amino acid deletions present in the *S. pombe* and *S. japonicus erh1* CDSs, respectively, and grey segments to indicate additional fragments present in some *ERH* CDSs; numbering is according to the human *ERH* CDS. Introns with the same phase and the same position in two or more CDSs are connected by dashed vertical lines. The *C. elegans* genes are represented by *erh-1* only since they share exactly the same positions for all their introns. The Ensembl (www.ensembl.org) identifiers for the sequences of the *ERH* genes are: *H. sapiens* (Ensembl vertebrates: ENST00000557016.6), *C. elegans* (Ensembl Metazoa: T21C9.4.1), *S. rosetta* (Ensembl Protists: EGD79537), *S. pombe* (Ensembl Fungi: SPAC19G12.17.1), *S. japonicus* (Ensembl Fungi: EEB06811), *D. discoideum* (Ensembl Protists: EAL70175), *A. thaliana* (Ensembl Plants: AT5G10810.1), *C. merolae* (Ensembl Plants: CMR260CT), *T. gondii* (Ensembl Protists: ESS33368) and *S. parasitica* (Ensembl Protists: KDO23370).

The number of introns within the coding sequence (CDS) of the *ERH* genes ranges from 0 to 6, with no intron in the *C. merolae ERH*, an organism whose genome is very compact and possesses only 26 protein-coding genes with introns (Figure 2) [8]. The *S. pombe* and *S. japonicus ERH* (*erh1*) genes possess, respectively, five and six introns and the first five of these introns in *S. japonicus* have the same positions as the *S. pombe* ones within the human *ERH,* CDS being the reference sequence in this comparison (in fact, the fifth intron in the *S. japonicus erh1* CDS is three nucleotides closer to the 5’ end due to the shortening of the immediately preceding exon when compared with the *S. pombe erh1*; for details, see [7]). The two *C. elegans ERHs* (*erh-1* and *erh-2*) have two introns each and both of them are in the same positions in their CDSs. Their first intron is a phase 1 type and shares the exact position with the third intron of these fission yeast species and with the second intron in the human *ERH* (within the triplet for glycine-31). This intron is also present in the *S. rosetta*, *D. discoideum* and *A. thaliana ERHs* (in all these species it is a phase 1 intron), while the *T. gondii* and *S. parasitica ERHs* lack it, so it seems to be common in *ERHs* from opisthokonts and absent from the *ERHs* from species belonging to the SAR supergroup (stramenopiles and alveolates, among others) [9]. The length of this intron is not conserved and ranges from 44 bp in the *C. elegans erh-2* to 7741 bp in the human *ERH*. The positions of two other introns of the human *ERH* are less conserved. The first of them, a phase 0 type, is placed immediately after the codon for the initial methionine and it is present in the exact position in the *D. discoideum* and *T. gondii ERHs*. It is also present in the *A. thaliana ERH*, albeit the N-terminus of its ERH was extended by six amino acid residues. In the *S. rosetta* gene, there is a phase 0 intron at the 5’ end but it sits after the codon for the proline residue placed immediately after the initial methionine. It is absent from the *ERH* genes in *C. elegans* and the fission yeasts. The third intron in the human *ERH* is a phase 2 type and sits within the codon for valine-71. In exactly the same position, there is also a phase 2 intron in the *D. discoideum*, *A. thaliana*, *T. gondii* and *S. parasitica ERHs*. In the majority of the rest of the compared *ERHs*, there are introns in this part of the CDS but none of them is a phase 2 type. One of them is the second intron in the *C. elegans* genes which is a phase 0 type and sits after the codon located three amino acid residues downstream of this valine. A possible explanation for this intron pattern is that all three introns in the human *ERH* were already present in the original gene and some of them, or even all, as in the case of the *C. merolae ERH*, have been lost or moved in some lineages.

The *ERH* gene is expressed in all major human adult and fetal normal tissues, which merits its inclusion in the group of housekeeping genes [10]. Even more so, the level of its expression does not change significantly between the tissues. There is only a 2.5-fold difference between the highest expression level observed in the pituitary gland and the lowest one in the peripheral blood leukocytes. In *S. pombe*, the gene is expressed at a similar level regardless of ploidy, growth stage and exposure to stress [7]. In *D. melanogaster*, two *e(r)* transcripts were observed due to the polyadenylation switching mediated by SXL, a master sex-switch protein [1,11]. The shorter transcript is present in adult flies of both sexes, whereas the longer one is found predominantly in the ovaries of mature females, although it is not translated. At least one of the transcripts, likely to be the longer one, is maternally deposited into the egg [12].

On the other hand, in the soybean *Glycine max*, *ERH* was reported to be induced in response to infection with pathogenic bacteria [13]. *ERH* is up-regulated in the electric organ of the mormyrid electric fish *Brienomyrus brachyistius* [14]. Increased expression of *ERH* was also detected during erythropoiesis [15] and in various human cancers, including breast, ovarian, liver, bladder, skin and gastric cancers [16,17,18,19,20,21]. (For more on the role of ERH in cancers, see the two reviews [22,23].) *ERH* is regulated post-transcriptionally by miR-574-3p in humans and the Musashi-1 protein in mice [24,25]. Both bind to the 3’ UTR of the transcript. The ERH protein level was significantly decreased in the murine *Notch2* null cells, thus, it seems that its gene is regulated by the Notch signaling pathway [26].

## 4. Structure of the Protein and Its Localization in the Cell

Indeed, comparison of amino acid sequences of the selected ERH proteins (Figure 1) shows that its sequence is highly evolutionarily conserved, especially in Vertebrata in which the *D. rerio* ERH differs from the human ERH by one conservative substitution only. In respect to the human ERH, the tunicate and sponge ERHs show 84% and 62% homologies, respectively. The two *C. elegans* ERHs (ERH-1 and ERH-2) display 53% and 37% homologies each to the human ERH and 39% between themselves. The *S. pombe* ERH (Erh1) shows 28% homology to the human ERH and 58% homology to the Erh1 protein from the other fission yeast species, *S. japonicus*. The latter displays the lowest homology (27%) to the human ERH among the species compared here. The two *P. mitrella* ERHs show 83% homology between themselves. Homology between the two ERHs from the fission yeasts seems to be low but it is in agreement with homologies between other orthologous proteins from these species (on average 55%), in which CDSs in protein coding genes are believed to evolve anomalously quickly [27]. This probably adds to about one billion years of the independent evolution of Fungi (the fission yeasts) and Metazoa (humans), resulting in considerable differences between their ERHs [28]. The homology between the two *C. elegans* paralogs is very low and indicates that one of them, ERH-2, could have been adapted for some additional function(s), but not in all nematodes, since some of them do not possess the *erh-2* gene [29]. Remarkably, the length of the ERH polypeptide chain has not changed during evolution. It ranges from 100 to about 110 aa with a typical length of 104 aa, which means that no domain has been added or deleted (Figure 1). Small differences in the length result from additional strings of amino acids at the N- or C-terminus (e.g., respectively, in *A. thaliana* and in *C. elegans*) and/or deletion(s) inside the chain (e.g., in the fission yeasts). Thus, almost all of the polypeptide chain of ERH constitutes the conserved core (from histidine-3 to leucine-95 in the human ERH). In particular, there are three highly conserved regions. The first one is the string of HTILLVQ in which three residues, H-3, L-7 and Q-9, are perfectly conserved in all the compared sequences, while I-5, L-6 and V-8 can be the conserved substitutions in some of these sequences. The second one includes six residues, R-17, Y-19, D-21, M-29, V-32 and Y-36, that can be the conserved substitutions in the other sequences. The third region contains two perfectly conserved residues, D-66 and Y-79, and five others, L-64, L-70, K-84, W-86 and I-87, that can be the conserved substitutions. The other highlight of the ERH sequence is that it is absolutely unique, i.e., there is no other known protein whose amino acid sequence is even distantly related to ERH. Therefore, its analysis provides no hint of its functions in the cell nor its localization.

**Figure 3:**
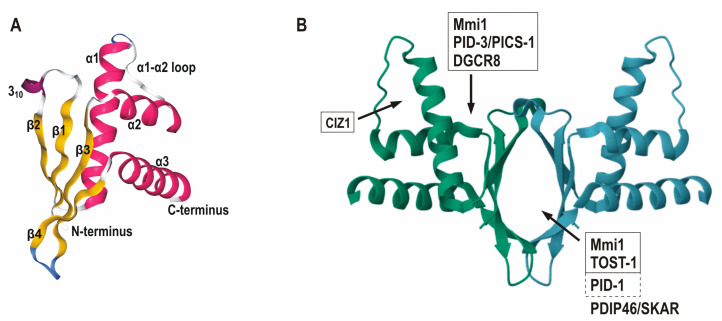
Three-dimensional structure of ERH and its protein partner binding sites. (**A**) The structure of the human ERH monomer (PDB ID: 2NML) is a screenshot from RCSB PDB (www.rcsb.org, accessed on 8 October 2023) and the secondary structures are shown in different colors (the α-helices α1, α2 and α3 with pink, the 3_10_-helix with violet, the β-strands β1, β2, β3 and β4 with yellow, and turns with blue). The α1-α2 loop and the N- and C-termini are also indicated. (**B**) The homo-dimeric assembly of the human ERH colored by chain (PDB ID: 2NML) is a screenshot from PDBe (www.ebi.ac.uk/pdbe, accessed on 8 October 2023). Arrows indicate the rough locations of the partner binding sites (only one from each pair of the binding sites is shown) and the ERH partners from different species are grouped together in boxes according to the binding sites based on the structural studies (the PDB identifiers for the structures with the partners are: 7CNC for the human ERH with DGCR8, 7X39 for the human ERH with CIZ1, 7O6N and 7EJS for the *C. elegans* ERH-2 with PID-3/PICS-1, 7EJO for the *C. elegans* ERH-2 with TOST-1 and 6AKJ for the *S. pombe* Erh1 with Mmi1). PID-1 competed with TOST-1 for the binding to ERH and PDIP46/SKAR interacted with ERH through the ERH homodimer interface, but these interactions have not yet been verified by crystal structures.

The three-dimensional (3D) structure of ERH has been resolved (Figure 3A). The human (PDB IDs: 1W9G [30] and 2NML [31,32]), murine (PDB IDs: 1WZ7 [33] and 1WWQ [34]), *C. elegans* (PDB ID: 7O6L [35]) and *S. pombe* (PDB ID: 6S2W [36]) ERHs in the free state were analyzed by X-ray crystallography and/or NMR spectroscopy and it was demonstrated that its folding is also conserved even between the human ERH and the *C. elegans* ERH-2 or the *S. pombe* Erh1, which show relatively low homologies at the amino acid level [35,36,37,38]. Unsurprisingly, its short polypeptide chain folds into a single domain. It has the β1-β2-α1-α2-β3-β4-α3 major secondary structure order with three amphipathic α-helices situated on one side (back side) of a four-stranded antiparallel β-sheet, which, according to the SCOPe database, constitutes a unique protein fold named ERH-like (Fold d. 330) belonging to the a+b (α+β) structural class. It was also named the RAGNYA fold [39]. There are also a very short 3_10_-helix between the first and the second β-strand and a flexible loop, up to 10 amino acids long, between the first and the second α-helix (α1-α2 loop). The deletions can shorten the length of this loop, as is found in ERHs from the two fission yeast species and ERH-2 from *C. elegans* (Figure 1). ERH can form the homodimer through the other side (front side) of the β-sheet from each monomer, with these β-sheets constituting an interface described as the β-sandwich or the pseudo-β-barrel (Figure 3B) [30,32]. Analysis of the 3D structures revealed that the majority of its most conserved amino acid residues are located on the β-sheet or immediately precede one of the β-strands, e.g., H-3, I-5, L-7, Q-9, R-17, Y-19, D-21, D-66, L-70 and Y-79 (positions in the human ERH) and are mainly involved in its dimerization, either through direct interactions or water bridges (I-5, L-7, Q-9, R-17, Y-19, D-21, L-70 and Y-79) [30,32,33,34]. Not surprisingly, the substitutions of I-11 and L-13 (I-5 and L-7, respectively, in the human ERH) with the bulky and positively charged arginine residues disrupted the homodimer of the *S. pombe* protein [36]. Thus, the formation of the homodimer is the most strictly conserved property of ERH and obviously crucial for its function in the cell. Interestingly, while the size and the 3D structure of this protein have been strictly maintained throughout the evolution of eukaryotes, experimental addition of an extra domain to its C-terminus (e.g., the EGFP protein) did not seem to change its behavior in the cell [10,40,41,42]. Similarly, while its amino acid sequence is also highly conserved, numerous amino acid substitutions (mainly to alanine) did not alter its behavior either [42]. Thus, the structure of ERH seems to be nearly “bulletproof”. Lastly, for all the above mentioned reasons, the 3D structures of ERHs from other species are expected to be predicted with very high accuracy using the AlphaFold software (current version 2.3.2) [43].

Studies on ERH in humans, *D. melanogaster*, *C. elegans* and *S. pombe* show that it localizes primarily to the nucleus [7,10,40,41,42,44,45,46]. This is consistent with the aforementioned lack of any known targeting signal in its amino acid sequence, as it is recognized that proteins smaller than ~40 kDa can also enter this cellular compartment by passive diffusion [47]. However, some data suggest that ERH may be able to enter the nucleus by piggy backing onto its protein partners possessing nuclear localization signals [48]. During the interphase, it seems to be present there mainly in a diffused pattern in the nucleoplasm, i.e., excluding nucleoli, and, in the human cell nucleus, it can form small bodies, some of which could be nuclear speckles and DNA replication foci [7,10,40,42,46]. In *S. pombe*, Erh1 also forms nuclear foci in vegetative cells and these foci seem to be the meiotic mRNA degradation sites which, upon entry into meiosis, converge to the single Mei2 dot [36,37,46]. During mitosis, when the nuclear envelope breaks down, ERH fills the entire human cell, excluding the space occupied by chromosomes [41,42]. However, there are reports showing that ERH could be detected also in the cytoplasm in *S. pombe*, *C. elegans* and humans without nuclear envelope breakdown [7,45,49].

## 5. Functions of the Protein

### 5.1. Data Inferred from Mutations and Silencing of the Gene

Since the amino acid sequence of ERH does not deliver any information about the role of ERH in the cell, other means of obtaining such knowledge should be sought. One possible approach is to analyze changes or pathological conditions that accompany mutations and silencing of *ERH*.

In *D. melanogaster*, the hypo-morphic mutation in the *e(r)* gene resulted in the augmentation of aberrations in the wing formation, but in the *r* mutant background only, and it decreased the level of the *r* transcript, even in the wild-type *r* background [1]. It was proposed that *e(r)* could have some regulatory function in the metabolism of pyrimidines [1]. The *e(r)* null mutants were also morphologically wild-type in the wild-type *r* background; however, both sexes exhibited decreased viability in addition to diminished fertility in females [12]. In the *r* mutant background, these mutations again enhanced the truncated wing phenotype [12]. Lastly, the *e(r)* null mutation drastically reduced the level of the E(spl) protein, the gene of which is regulated by the Notch signaling pathway [44].

In *S. pombe*, the disruption of the *erh1* gene is not lethal but leads to various defects during the vegetative growth, including sensitivity to lower temperatures, sorbitol, SDS and hydroxyurea (the last of which depletes dNTP pools causing DNA replication stress [50]), delayed recovery from the stationary phase and enhanced arrest in the G1 phase following nutritional stress [7,36,37,46,51]. Moreover, the accumulation of some meiotic mRNAs that are normally selectively eliminated during this stage of the life cycle and some ncRNAs that are regulated environmentally or developmentally, and defects in the facultative heterochromatin assembly, mainly at meiotic loci and the *rDNA* repeats, as well as defects during the sexual differentiation and meiosis, including lack of the Mei2 dot and reduced mating/sporulation efficiency, were observed [36,37,46,51].

Interestingly, in *Saccharomyces cerevisiae*, which does not possess the *ERH* gene in its genome, the expression of the human *ERH* resulted in enhanced pseudo-hyphal growth [32].

The *C. elegans erh-2* mutant was sterile, exhibited abnormal chromosome segregation and cell division during embryogenesis, and had decreased levels of mature piRNAs (21U-RNAs) with concomitant accumulation of their precursors [29,45].

To date, no pathological mutation in *ERH* is known in humans. There is one report on the chromosomal deletion of the region with *ERH* that was associated with mild intellectual disability, congenital heart defects and brachydactyly, among others, in three children, but this haploinsufficiency cannot be attributed exclusively to this gene since the deletion encompassed at least 18 additional protein coding genes [52]. According to UniProt, there are over 20 SNPs within the CDS (UniProt ID: P84090), mostly in its second half, that change amino acid residues, of which, however, none is of any known significance.

Silencing of *ERH* resulted in the inhibition of cell proliferation in cervical, ovarian, bladder and skin cancer cell lines [17,19,20,41]. It also decreased viability in the colorectal cancer cell lines as well, and the effect was greater in the cells harboring the *KRAS* mutation [53]. The silencing also promoted apoptosis, suppressed migration and invasion, and reversed the levels of the epithelial–mesenchymal transition protein markers in the ovarian and bladder cancer cell lines [17,19,54]. It led to hypersensitivity to hydroxyurea and other replication stress-inducing agents, and impaired recovery following replication stress and DNA replication per se in the bone cancer cell line, and diminished the capability of DNA damage repair following UV irradiation in the liver cancer cell line [18,55]. Low *ERH* expression was associated with better survival of colorectal cancer and lung cancer patients with tumors harboring *KRAS* mutations (though not significantly in the latter patients) [53]. Similarly, ovarian cancer patients with lowered *ERH* expression had better survival [17]. Decreased *ERH* expression also correlated with better survival of gastric cancer patients, albeit not significantly, and another study showed the opposite in the case of patients with this cancer having an elevated level of the ERH protein [21,56]. Overexpression of *ERH* augmented the apoptotic effect of anthocyanins on the gastric cancer cell line [57].

Silencing of *ERH* resulted in the loss of the mitotic kinesin CENP-E at kinetochores of mitotic chromosomes leading to lack of the proper kinetochore–microtubule attachment and thus to a severe misalignment of chromosomes [41,53]. This effect was due to a drastically decreased level of CENP-E, which seemed to be the result of a reduced level of the *CENPE* mRNA. Interestingly, this decrease was not the aftermath of less *CENPE* transcription per se, but resulted from a defect in the splicing of the pre-mRNA [53]. Likewise, splicing of pre-mRNA of the *ATR* gene that codes for a kinase involved in the response to DNA damage and its repair was affected by the silencing of *ERH* and resulted in a reduced level of the kinase in cells and its diminished localization to the chromatin following replication stress [18,55]. Two of these studies showed that the silencing of *ERH* led to the deregulation of even more genes in humans. The first pointed to only 87 genes but, within this limited set, revealed an enrichment for genes that, like *CENPE*, are involved in the cell cycle, as well as for those that, like *ATR*, are involved in the DNA replication and/or DNA repair [53]. The other study indicated an even greater effect, since the levels of about 1500 transcripts were affected [55]. Among the down-regulated genes were those involved in the cell cycle, chromatin organization or DNA metabolism, particularly those related to the DNA replication and DNA repair, and among about 750 genes exhibiting the defective pre-mRNA splicing, those engaged in chromatin organization or DNA metabolism were enriched.

Silencing of *ERH* also up-regulated many human orthologs of the *S. pombe* meiotic genes and, more generally, derepressed spermatogenic and oogenic genes, as well as other lineage-specific genes, e.g., hepatic ones, and repetitive elements like endogenous retroviruses and the short interspersed nuclear elements (SINEs), but not the long ones (LINEs) [58]. Moreover, a decreased level of trimethylated lysine-9 on histone H3, a methylation which is one of the hallmarks of the mammalian heterochromatin, was observed in the domains containing protein coding genes and especially in ones with pseudogenes and satellite repeats. At the same time, the *rDNA* repeats were unchanged. In accordance with this, the abundance of the histone 3 lysine-9 *N*-methyltransferase SUV39H1 on heterochromatin was also decreased [58].

Lastly, it was reported that the silencing of *ERH* also resulted in changes in post-translational modifications of some nuclear proteins in human cells, namely decreased phosphorylation of the major substrates of the SRPK1 kinase, lamin B receptor that tethers heterochromatin to the nuclear periphery, among others, and the SR splicing factors (SRSFs) [49,59,60].

Taken together, these reports suggest that ERH may be necessary for the maintenance of the proper expression levels of the subset of genes that are required for the cell cycle, DNA replication and DNA repair, and that could be achieved by the action of ERH on the pre-mRNA splicing in a positive manner. This is in agreement with the increased *ERH* expression level observed in cancers and the inhibition of cell proliferation in cancer cell lines by the silencing of *ERH*. Specifically, these genes could be involved in DNA damage response, as it needs the coordination of the cell cycle and the DNA replication with DNA repair. Moreover, it is conceivable that the observed effects of the *e(r)* mutations on the expression of the *D. melanogaster r* gene and the level of the E(spl) protein resulted also from the defect in the splicing of their transcripts. Apart from the decreases in the protein levels in the cell, which are secondary effects, it seems that ERH can probably also indirectly alter the level of phosphorylation of several proteins, some of which play an important role in pre-mRNA splicing, suggesting how ERH could modulate this process. However, ERH appears to be involved in the metabolism not only of mRNA but also other RNA types, like piRNA and some other ncRNAs, as well as in DNA replication, and in the formation of the heterochromatin at protein coding loci and repetitive element loci to repress them.

### 5.2. Data Inferred from Partners of the Protein

Another approach to establishing the role of ERH in the cell is to discover its interactions with partners with known functions in order to obtain a hint of the processes in which ERH could be involved. Although ERH was suggested to be an RNA-binding protein, all of its molecular partners identified so far have turned out to be proteins [61,62].

Initially, it was ERH that was identified as a binding partner during studies devoted to other proteins. The first of the partners was discovered in the claw frog *Xenopus laevis* [63]. In the yeast two-hybrid screen for molecular partners of DCoH/PCD, the frog ERH (100% identical with the human ERH at the amino acid level) was demonstrated to interact with it directly. DCoH/PCD is a coactivator for the HNF1 transcription factors (acting as a dimerization cofactor for HNF1s) and it was suggested that ERH could act as a transcriptional repressor that interferes with the enhancement of the HNF1-dependent transcription by DCoH/PCD via binding to the latter. Later, this putative action of ERH has often been used for the interpretation of results. Next, in the search for proteins associated with the human SPT5, apart from its hetero-dimerization partner SPT4 with which it forms a transcription factor that can regulate RNA polymerase II during the elongation stage, the protein arginine methyltransferase PRMT5 and its substrate adaptor pICln (currently CLNS1A), ERH was detected using the affinity purification followed by the mass spectrometry (AP-MS) method [64,65,66]. SPT5 was modified by PRMT5 generating a symmetrically dimethylated arginine residue, but it could also associate with and be dimethylated by another arginine methyltransferase, PRMT1 generating asymmetrical dimethylarginines, and both types of methylations regulate the association of SPT5 with RNA polymerase II and the effect of SPT5 on the transcriptional elongation [64,66,67]. No attempt to demonstrate a direct interaction between ERH and any of the other proteins was reported. Interestingly, during the search for proteins that associate with the human CHTOP (also known as FOP or SRAG), a chromatin-associated protein that is involved in transcriptional regulation, both PRMT1 and PRMT5, the methylosome component MED50 (currently WDR77) directly interacting with PRMT5 and ERH, were again identified by AP-MS [66,68,69,70]. CHTOP could bind to 5-hydroxymethylocytosine in DNA and recruited both methyltransferases. PRMT1 dimethylated CHTOP, and both enzymes could dimethylate arginine-3 on histone H4, but it is the action of PRMT1 that mainly activated the transcription of several cancer-related genes [68]. No proof for a direct binding of ERH by any of these proteins was delivered. Likewise, in search for proteins associated with the human FCP1, a phosphatase specific for the carboxyl-terminal domain (CTD) of the large subunit of RNA polymerase II and required for the transcription elongation, PRMT5, MED50 and ERH were again detected using AP-MS, together with the RPB2 subunit of this polymerase and the kinase NDR1 [71,72]. Similarly, FCP1 was dimethylated by PRMT5 [71]. However, functional consequences of this modification and an explicit proof for a direct interaction of ERH with the other proteins were not delivered.

In the first attempt, specifically designed to find binding partners of the human ERH, two nuclear proteins, PDIP46/SKAR and CIZ1, were identified by the yeast two-hybrid screen as interacting with it directly [10,40]. CIZ1 was originally discovered as a molecular partner of p21 (currently CDKN1A), a universal inhibitor of cyclin-dependent kinases (CDKs) [73,74]. p21 binds to the region in the central part of CIZ1 and CIZ1-p21 complex was reported to be exported from the nucleus to the cytoplasm [74]. This led to the hypothesis that CIZ1 could be involved in the regulation of the cellular localization of p21. However, later reports showed that CIZ1 occupies the entire nucleus with the exception of the nucleoli and with its concentration in replication foci or factories, in which it is involved in the initiation of the nuclear DNA replication, acting at the assembly of the replication complex at the origin of replication [40,73,75]. Its N-terminal half possessing DNA replication activity binds cyclin A-CDK2 complex and is phosphorylated by this kinase [73,76,77]. Its C-terminal third can bind to the nuclear matrix, a framework upon which chromatin is immobilized and which also organizes DNA replication factories by the recruitment of the origins of replication [73,78]. ERH and p21 bind to the same region in CIZ1, therefore they could compete for the binding to this protein [40]. CIZ1 recruited ERH to replication foci and it was established that the amino acid residues in ERH that are critical for this interaction and recruitment are situated just before and within the α1-α2 loop [40,42]. It was suggested that the CIZ1-deficient cells exhibit a defect in DNA repair following exposure to hydroxyurea and the CIZ1-immunodepleted extract showed a significantly reduced capacity in the DNA repair assay [79,80]. CIZ1 can also bind to DNA and is involved in the enhancement of the estrogen receptor α (ERα) transcriptional activity [81,82]. Lastly, CIZ1 is required for the localization of the Xist lncRNA at the inactive chromosome X by the anchoring of Xist to the nuclear matrix [83].

PDIP46/SKAR (also known as POLDIP3, currently PDIP3) resides in the nucleus, with the exception of the nucleoli and with its highest concentration in nuclear speckles [10,42,84]. It was originally discovered as a molecular partner of the p50 subunit of DNA polymerase δ, a main elongation enzyme during nuclear DNA replication [84,85]. PDIP46/SKAR, DNA polymerase δ and MCM2, a helicase subunit that is present at the origin of replication, were found on chromatin in close proximity to one another [86]. PDIP46/SKAR facilitated DNA synthesis by DNA polymerase δ on complex templates and could interact with PCNA, a processivity factor for DNA polymerases δ and ε [86]. Recently, it was demonstrated that PDIP46/SKAR (as POLDIP3) is involved in the responses to replication stress [87,88]. It facilitates the activation and maintenance of the DNA damage checkpoint and associates with the RTEL1 helicase and together they are needed for the prevention/resolution of the R-loops ahead of the replication forks. The protein was rediscovered as SKAR and it was shown to participate in the pre-mRNA splicing as a component of the exon junction complex (EJC) forming on a (pre-)mRNA molecule following intron removal [89,90,91]. ERH was also identified as a component of the EJC interactome [92]. PDIP46/SKAR could bind to S6K1 and be phosphorylated by that kinase [89]. It is possible that ERH and S6K1 compete for the interaction with PDIP46/SKAR, since their binding regions overlap in the C-terminal half of the protein [10]. PDIP46/SKAR recruited ERH to nuclear speckles and it was shown that the amino acid residues in ERH that are essential for interaction and recruitment are located on the surface of the four-stranded β-sheet [42]. Since these surfaces constitute the homodimer interface, it was initially suggested that PDIP46/SKAR can interact with the ERH monomer only [42]. However, later structural studies on ERH and its partners, like Mmi1 and TOST-1, demonstrated that they can bind to the ERH homodimer interface without interfering with its dimerization [37,38] (see also below). The human PDIP46/SKAR was also identified as a component of the conserved transcription and export complex (TREX) that functions in mRNA export to the cytoplasm [93,94]. The recruitment of TREX to a (pre-)mRNA is splicing-dependent and PDIP46/SKAR (as PDIP3) seems to be involved in the release of polyA^+^ RNA from nuclear speckles [94,95]. Interestingly, ERH and CHTOP (as SRAG) were also reported to be components of this complex [93]. The function of ERH in the export has not been determined yet but the methylation of CHTOP and the other component of TREX, ALYREF by PRMT1, promoted the handover of mRNA to the mRNA export receptor NXF1 [96].

Unfortunately, the significance of the interaction of ERH with either PDIP46/SKAR or CIZ1 has not been studied further. Nevertheless, the binding of the latter and the key ERH amino acid residues for this interaction were also recently confirmed at the structural level (PDB ID: 7X39) [97]. ERH and CIZ1 (its fragment tethered to ERH via a linker) form a hetero-tetramer with 2:2 stoichiometry and, interestingly, for the binding of the CIZ1 fragment by the negatively charged V-shaped cleft formed by the α1, α2 and α3 helices and the α1-α2 loop in ERH (Figure 3B), it adopts the additional β-strand within this loop, which forms, along with two β-strands of CIZ1, the intermolecular antiparallel β-sheet [97].

ERH was detected as a hit in a large interactome of SMN, a protein that is crucial for the deposition of the hetero-heptamer comprised of the Sm proteins on snRNAs, to form the snRNP core particles in the cytoplasm, which are then imported to the nucleus for the assembly of the spliceosome complex [98,99,100]. When SMN enters the nucleus, it is present there in Cajal bodies and gems [99,101]. Moreover, SNRPD3, itself one of these Sm proteins, was reported to be a molecular partner of ERH [53]. Although direct interactions between ERH and SNRPD3 or SMN were not demonstrated, it was suggested that ERH could be a part of the complex that is necessary for the splicing of the pre-mRNA from the *CENPE* gene, among others [53]. Interestingly, the formation of the snRNP core particles depends on the methylation of arginine residues in three Sm proteins, SNRPDB/B’, SNRPD1 and this SNRPD3, by the PRMT5 methylosome (the PRMT5:MED50/WDR77 hetero-octamer) assisted by the substrate adaptor pICln/CLNS1A [66,100,102]. This modification increases their binding to SMN, which contains the Tudor domain recognizing the symmetrically dimethylated arginine residue [103]. Moreover, SMN is also required for the resolution of the R-loops in transcription termination regions when, through its Tudor domain, it is recruited by the PRMT5-generated dimethylarginine residue in CTD of the large subunit of RNA polymerase II [104].

From the longest list of the protein partners of the human ERH that has been reported so far, PDIP46/SKAR, CIZ1, CHTOP and PRMT1 were already known [55]. Three novel ones, THRAP3, BCLAF1 and C1QBP, are other proteins involved in the mRNA processing, and two proteins, DGCR8 and DROSHA, are involved in the miRNA processing [55]. For some of these partners, the necessity for the presence of RNA in forming complexes with ERH was excluded, but the participation of third proteins was not [55]. Specifically, THRAP3 (also known as TRAP150) and BCLAF1 are involved in the splicing of the pre-mRNA and the nuclear export of the mRNA of *ATM*, a gene coding for another kinase involved in DNA damage repair [105]. They are components of the EJC interactome and, together with ERH, they might form the hypothetical ternary complex named BET [92,106]. THRAP3 is also modified by PRMT5 [102]. As for the miRNA processing, recent reports show that ERH indeed interacts directly with DGCR8, which is also a direct partner of DROSHA and forms with it Microprocessor, a heterotrimeric complex comprised of one DROSHA and two DGCR8 molecules, which is responsible for the first step in miRNA maturation [107,108]. ERH seems to be an additional component of Microprocessor, required for the processing of the suboptimal miRNA hairpins [107,109]. ERH and the free fragment of DGCR8 form a hetero-tetramer with 2:2 stoichiometry and the DGCR8 fragment binds to the hydrophobic groove between the α1 helix and the β-sheet (PDB ID: 7CNC) (Figure 3B) [107]. Thus, the full Microprocessor seems to be a hetero-pentameric complex [107].

Direct interactions between the human ERH and SAFB1 or SAFB2, paralogous components of the nuclear matrix, were also reported [49]. SAFB1/2 populate the nucleus with the exception of the nucleoli and concentrate in nuclear speckles, and bind the SRPK1 kinase, thereby repressing its activity [49,110]. SAFB1 binds to the scaffold/matrix attachment regions in DNA, cooperates with the MajSat RNAs in the stabilization of the heterochromatin architecture, may participate in the chromosome X inactivation mediated by Xist, and is a component of the DNA damage response [49,111,112,113]. ERH interacted with the same part of SAFB1/2 as SRPK1 and this binding liberated SRPK1 and reversed the inhibition exerted by SAFB1/2 on the kinase [49]. Interestingly, recently SAFB2 was shown to be an accessory protein of Microprocessor, which also enabled the processing of the suboptimal miRNA hairpins [114]. However, SAFB2 did not interact with Microprocessor through binding to ERH, but could directly bind to the DROSHA component [114]. SAFB1/2 are also involved in the repression of the ERα transcriptional activity but ERH did not seem to interfere with this SAFB1/2 function [49].

Other reported partners of the human ERH are the tumor growth inhibitor HOTS, the transcription factor STAT3, the tumor suppressor p53, the Mediator subunit MED31, the transcriptional corepressor TLE1, the heat shock protein HSPA8, the histone 3 lysine-9 *N*-methyltransferase SETDB1, the SRC-type kinase activator UNC119, the RNA-binding protein DKFZP564O0523, the GTPase-activating protein DEPDC1B, the uncharacterized protein FAM208B, the mTOR signaling inhibitor DDIT4, the ribosome 40S subunit protein RPS3, the eukaryotic initiation factor 2 subunit EIF2α, the phosphodiesterase SMPDL3A, and the nucleolar zinc-finger protein ZNF330/NOA36 [21,48,55,115,116,117,118,119,120]. ERH was also reported to cofractionate with histones but in an RNA-dependent manner [58].

In *D. melanogaster*, only two proteins have been found so far to interact with its ERH. One of them is again RPS3 and the other is the ribosome 60S subunit protein RPL19 [48]. Both of them interacted with this ERH directly and RPS3, as well as its human ortholog, were suggested to piggy-back the *D. melanogaster* and human ERHs into the nucleus [48]. The *D. melanogaster* ERH was phosphorylated by CK2 in vitro [5] and the follow-up study with mutants showed that the phosphorylation at either of the two highly conserved CK2 sites might generate the biologically active protein, whereas the unphosphorylated or doubly phosphorylated protein would be inactive [121]. Based on the crystal structure of the human ERH, it was proposed that the phosphorylation could regulate the dimerization and interaction with binding partners [30].

None of the human protein partners of ERH have been reported to interact with any of the two ERH paralogs in *C. elegans*. Instead, ERH-2 binds directly to PID-3/PICS-1 and together they form a stable hetero-tetramer with 2:2 stoichiometry [29,35,38,45]. The PID-3/PICS-1 fragment (free or tethered to ERH via a linker) binds to the same hydrophobic groove between the α1 helix and the β-sheet in ERH as DGCR8 does (PDB IDs: 7EJS and 7O6N) (Figure 3B) [35,38]. The ERH-2:PID-3/PICS-1 hetero-tetramer with two molecules each of TOFU-6 and IFE-3 (one of the *C. elegans* homologs of the mRNA cap-binding protein eIF4E) form a hetero-octamer with 2:2:2:2 stoichiometry named the PETISCO complex, in which TOFU-6 connects PID-3/PICS-1 and IFE-3 [29,35]. PETISCO interacts with its effector proteins, PID-1 and TOST-1 [29,35]. Their free fragments bind to ERH-2 in a mutually exclusive manner using the homodimer interface which is located opposite to its PID-3/PICS-1 binding site, thus the ERH-2 dimer could simultaneously bind two molecules of PID-3/PICS-1 and two molecules of either PID-1 or TOST-1 (PDB ID: 7EJO for the TOST-1 fragment tethered to ERH via a linker) (Figure 3B) [29,35,38]. The hetero-octamer, comprising two molecules each of TOFU-6, PID-3 and ERH-2 (i.e., PETISCO lacking two molecules of IFE-3) and two molecules of either TOST-1 or PID-1, is known as the PICS complex [45]. In the germline cells, the complex with PID-1 is largely concentrated at the perinuclear zone, i.e., outside the nucleus, and is required for piRNA maturation, albeit in a not yet established manner [45]. During embryogenesis, the complex with TOST-1 accumulates in the nucleus and is needed for chromosome segregation, which is not dependent on piRNA biogenesis [45]. Interestingly, ERH-1 does not bind to PID-3/PICS-1 but it can interact with either of the two PETISCO/PICS effectors, PID-1 and TOST-1 [35].

The first partner of Erh1 discovered in *S. pombe* was Mmi1, a foci-forming nuclear protein that promotes the selective elimination of the meiosis-specific transcripts and the down-regulation of Mei2, a master regulator of meiosis, during vegetative growth [36,37,46,122,123,124]. Mmi1 also causes the retention of introns in the meiotic transcripts [125]. Erh1 and Mmi1 form a hetero-tetramer with 2:2 stoichiometry named the EMC complex, and ERH binds the Mmi1 fragment (tethered to ERH via a linker) using two sites, one that overlaps with the TOST-1 and PID-1 binding site and the other that overlaps with the PID-3/PICS-1 and DGCR8 binding site (PDB ID: 6AKJ) (Figure 3B) [37,46]. (For a more detailed comparison of the binding sites in ERHs, see [97,126].) EMC can associate with one of two complexes in the vegetative cells [46]. Together with MTREC, the Mtl1-Red1 core complex, they promote the degradation of the meiotic mRNAs by the recruited nuclear exosome and facilitate the assembly of the heterochromatin islands at the meiotic genes mediated by chromatin-associated RNAs [46]. The physical connection between Mmi1 and the nucleus-specific exosome component Rrp6 seems to be mediated by Red1 [127,128]. EMC also prevents nuclear export of the meiotic mRNAs [37]. EMC can also cooperate with CCR4-NOT, a conserved multiprotein complex with various functions, to form another class of the facultative heterochromatin, the heterochromatin domains (HOODs) [46]. However, it was reported that CCR4-NOT is also necessary for the formation of the heterochromatin at the meiotic genes [129]. Erh1 and CCR4-NOT were also required for the assembly of the heterochromatin at the *rDNA* repeats to ensure its integrity [46]. Thus, in the vegetative cells, one way or the other, Erh1 seems to be involved in the formation of the several types of the facultative heterochromatin, which in the fission yeast is preferentially enriched for histone H3 with dimethylated lysine-9 [130]. In particular, Erh1 prevents the untimely expression of the meiotic genes by promoting both the assembly of the heterochromatin at their loci and the decay and nuclear retention of the transcripts in the non-meiotic cells. In turn, independently of Erh1, Mmi1 can directly interact with CCR4-NOT to accelerate the deadenylation of the artificial RNA substrate by the recombinant complex and can promote the ubiquitinoylation of Mei2 by the complex and the down-regulation of that protein [124,131]. Interestingly, Mei2 can interact with Erh1 directly and this could be necessary for the formation of the Mei2 dot in the meiotic cells [132]. The binding site for Mei2 in Erh1 has not been established yet but, since Mmi1 is also present in this dot and might form EMC, it seems that the *S. pombe* ERH (Erh1) could also interact simultaneously with two partners there [122,132]. Mei2 and Mmi1 also interact indirectly via binding to some lncRNAs, including meiRNA encoded by the *sme2* gene, at which locus the dot is formed [133,134,135].

Currently, no partner of ERH has been reported in plants.

The small protein has surprisingly many partners, albeit only some of them have been proven to show direct interaction. Particularly, there are many in vertebrates, which are a group with astonishingly conserved ERH, and the plethora of partners can be the very reason for this conservation, i.e., the necessity for the maintenance of interactions with so many partners has kept its amino acid sequence unchanged, as PDIP46/SKAR, CIZ1 and DRGC8 bind to three distinct sites in the human ERH (Figure 3B). Moreover, although ERH is conserved remarkably, this does not apply to most of its partners. On the contrary, some of them are unique for each taxon. Mmi1 seems to be restricted to the fission yeasts and Mei2, while present also in plants, is absent from animals [136]. PID-3/PICS-1, TOST-1 and PID-1 are present in nematodes only [35]. CIZ1, PDIP46/SKAR, DGCR8 and SAFB1/2, to consider only the positively verified direct partners, seem to be restricted to animals, in which, according to Ensembl, they are quite conserved in vertebrates and, according to UniProt, their homologs are present in *D. melanogaster* and, at least in the case of DGCR8, also in *C. elegans*. Nevertheless, there are some similarities between the identified ERH partners. Many of them are concentrated in various nuclear bodies, e.g., Mmi1, Mei2, CIZ1, PDIP46/SKAR and SAFB1/2 [49,75,90,111,122,133]. They share also some structural features, like the ability of PID-3/PICS-1, DGCR8 and SAFB1/2 to form homodimers by themselves [35,49,107,114], the presence of an intrinsically disordered region(s) in Mmi1, TOST-1, CIZ1, DGCR8, SAFB1/2, THRAP3 and BCLAF1 [37,49,74,97,106,107], and the occurrence of the RRM domain(s) that can bind an RNA and/or a protein partner in Mei2, PID-3/PICS-1, PDIP46/SKAR and SAFB1/2 [35,49,89,114,132]. Both CIZ1 and SAFB1/2 can bind to DNA and the nuclear matrix [49,78,81,114]. Likewise, some proteins from the ERH interactome possess the Tudor domain that binds a symmetrically dimethylated arginine residue, e.g., SMN and TOFU-6 [35,103].

Obviously, all these features are not limited to the identified ERH partners. Particularly in mammals, there is the YTH domain-containing protein family with limited similarity to Mmi1 [137]. The family member YTHDC1 (YT521-B) also forms multiple nuclear foci (YT bodies) and possesses the region encompassing the YTH domain with 24% identity to the region with YTH in Mmi1 [122,137]. This domain is used by Mmi1 to bind the unmethylated hexameric UU/C/GAAAC motif present in the meiotic mRNAs and also in several lncRNAs, and is dispensable for its interaction with Erh1 but, in the mouse embryonic stem cells, YTH of YTHDC1 recognizes the chromatin-associated retrotransposon transcripts hallmarked with the monomethylated adenosine (m^6^A) and is involved in the formation of the heterochromatin at their loci [37,122,125,130,138]. Interestingly, this process requires the *N*-methyltransferase SETDB1 that was reported to interact with the human ERH, albeit it generates the trimethylated lysine-9 on histone H3 instead of the dimethylated one that was associated with the Erh1 activity in *S. pombe* [46,116,130]. YTHDC1 also recognizes m^6^A residues on Xist and promotes the silencing of the gene transcription on the chromosome X mediated by this RNA [139]. Moreover, there are orthologous MTREC (PAXT) and CCR4-NOT complexes in humans [131,140]. On the other hand, CCR4-NOT and a YTH domain-containing protein are present in *S. cerevisiae*, while this yeast lacks MTREC and the *ERH* gene [131,140,141].

**Figure 4:**
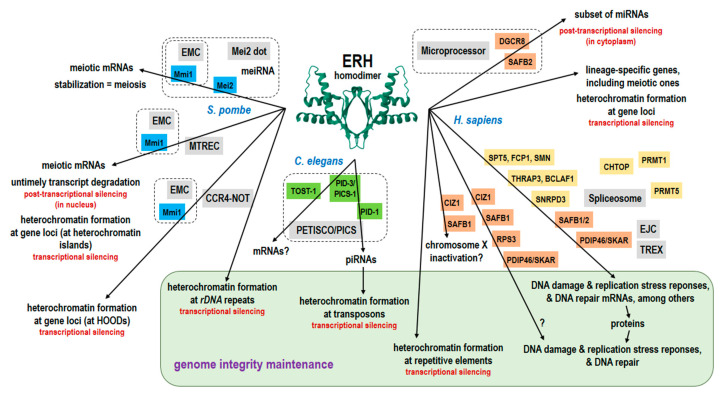
Diagrammatic summary of the ERH functions, including the proposed genome integrity maintenance. Question marks indicate processes in which the involvement of ERH has not been proven. The ERH icon was downloaded from PDBe (www.ebi.ac.uk/pdbe, accessed on 20 August 2023). The names of the ERH partners are on colored backgrounds according to the species (in *S. pombe* blue, in *C. elegans* green, and in *H. sapiens* dark orange for the direct binding partners and light orange for those that have not been demonstrated to interact with ERH directly). The names of the complexes are highlighted in grey and these comprising ERH are outlined with a dashed line.

In the fission yeast, ERH is needed for the degradation and the prevention of the nuclear export of the meiotic mRNAs (Figure 4). In the nematode, it is required for piRNA biogenesis, and in humans, it is involved in the biogenesis of mRNAs and its export and the biogenesis of miRNAs (besides the repression of the lineage-specific genes and the repetitive elements). Thus, one could suggest that, during the evolution of eukaryotes, ERH has been adopted for the needs of the different processes. In line with this, the evolution of the ERH amino acid sequence has been driven by adaptation to the protein partners as evolutionary novelties. On the other hand, these sets of the ERH partners which are not overlapped could be explained, at least in part, by coincidence only, i.e., the differences in the order of their identification in these organisms. After all, the processing of mRNA and the formation of heterochromatin belong to processes commonly found in eukaryotes. It is worth noticing that a deletion in *erh-2* in *C. elegans* and the silencing of *ERH* in humans led to the similar phenotype, the abnormal chromosome segregation (lagging chromosomes), and the *C. elegans* TOST-1 might play a role in the splicing [41,45,53]. Besides, a role for the other paralog, ERH-1 has not yet been established. Likewise, ERH is involved in the formation of the heterochromatin in *S. pombe* and in humans, but in the latter no specific partner has been demonstrated. Thus, we do not yet know all the ERH partners (or we are unaware of all the activities of those already known). This applies not only to plants, as mentioned earlier, or to *C. elegans,* but also to the rest of the above mentioned organisms, and in particular to *D. melanogaster*, a very convenient model for such studies, in which so far only two partners have been identified experimentally [48]. Surprisingly, both are ribosomal proteins indicating another role for ERH, albeit not necessarily in the translation in the cytoplasm, since these proteins are present in the nucleus, where the ribosome subunits are assembled, and RPS3 can also act as a DNA repair enzyme [142,143]. Moreover, several other homologs of the human ERH partners seem to be present in the *D. melanogaster* proteome and its ERH was functionally replaced by the human ERH [48]. Nevertheless, there are partners that occur only in a single taxon, like those in nematodes, so some processes confined to roundworms should exist. Indeed, while piRNAs function in animals from hydra to humans, *C. elegans* uses a unique pathway for their biogenesis [144,145]. It is possible that this is the very reason for the possession of the additional, atypical ERH paralog.

Regardless of whether we already know all the ERH partners, the quite extensive knowledge of the ERH interactome has confirmed the findings from studies with the mutations and the silencing of *ERH,* while delivering data heading towards its additional functions. ERH seems to be a component of several key complexes involved in nucleic acids’ metabolism, albeit the direct partners that ERH interacts with within some of them are not yet known. In particular, in humans, ERH could be involved not only in pre-mRNA splicing, but also in the initiation, elongation and termination of the transcription (producing pre-mRNA), and in the export of mature mRNAs to the cytoplasm, and these processes are accompanied by the dimethylation of arginine residues. Furthermore, ERH is required for the biogenesis of miRNA.

## 6. Functions of the Protein: Conclusions

ERH is a very small, single domain and globular protein with a unique three-dimensional structure that is tightly packed with the secondary structures, with the exception of one disordered region, the short loop. Its structure, probably invented at the beginning of the evolution of eukaryotes, has stayed exactly the same up to humans, including the size (in no lineage has ERH acquired any additional domain); however, it is not essential in simple eukaryotes, since in some lineages its gene has been lost and the deletion of *erh1* in *S. pombe* is not lethal. The protein is mainly nuclear, but can also be found in the cytoplasm. It has identified neither enzymatic activity nor RNA-binding ability and its only known biochemical action is oligomerization, i.e., its homodimerization that forms the scaffold for the building of the higher-order oligomers. ERH has at least three discrete binding sites for its partners. Surprisingly, the binding to one of these sites, which is near the homodimer interface, does not require the dissociation of the homodimer, and following the binding to the second site, the additional β-strand can be adopted in its loop. ERH could bind two partners simultaneously, e.g., in PETISCO/PICS or in EMC with Mei2, but sequential binding cannot be excluded. There can be competition between ERH and other proteins for binding to ERH partners, so its binding to these partners could liberate the other protein and allow it to execute its function, e.g., the SRPK1 kinase to phosphorylate SR proteins, the S6K kinase to phosphorylate its other substrates, or p21 to bind to the complex of cyclin and cyclin-dependent kinase.

The disparate protein partners in *S. pombe*, *C. elegans* and humans can be partially explained by the involvement of ERH in the biogenesis of the different types of RNA: mRNA, piRNA, miRNA, and ncRNA, other than the two latter types, in these organisms. However, a common theme regarding all these RNAs is the fact that they all are RNA polymerase II transcripts. In humans, ERH seems to be involved in all stages of mRNA biogenesis in the nucleus, including its export, and particularly in splicing, but only for the subset of mRNAs, mainly of DNA metabolism, chromatin organization and cell cycle genes, along with the processing of the polycistronic transcripts which harbor the suboptimal miRNA hairpins. Likewise, in *S. pombe*, Erh1 seems to be required for the degradation of the subset of RNAs, namely the meiotic mRNAs and some ncRNAs. Thus, although in humans and *S. pombe ERH* seems to be a housekeeping gene, ERH is not a general factor but rather an auxiliary one in the biogenesis of some RNA polymerase II transcripts. Interestingly, many stages of the biogenesis of mRNA and its export are accompanied by the dimethylations of arginine residues by PRMT1 and/or PRMT5. It is also worth noticing that, while Erh1 in *S. pombe* is necessary for the post-transcriptional silencing of the meiotic genes within the cell nucleus and the other ERH-dependent transcripts, like miRNAs, are also involved in the silencing at this level, they do it in the cytoplasm. Lastly, ERH partners in plants have not been identified yet, but one can speculate that, besides those involved in the biogenesis of mRNA, those partners could be some of the proteins required for the biogenesis of siRNA, as this type of RNA is involved in post-transcriptional silencing in plants [146]. On the other hand, Mei2 homologs are present in plants, so ERH could act there, as in *S. pombe*, i.e., to control meiotic genes, albeit in a modified manner, since Mmi1 seems to be restricted to the fission yeasts.

In humans, ERH depletion led to the accumulation of the incompletely spliced pre-mRNAs of DNA damage response (e.g., *ATR*), cell cycle (e.g., *CENPE*) and chromatin organization genes. On the other hand, the retention of introns is an additional stage in the expression of some genes, which enables the storage of almost fully spliced transcripts in the nucleus, ready for the activation of the removal of the remaining intron(s) by appropriate signals (some of them rather constitutive, and known as detained introns [147]). Such delayed expression transcripts could be useful in case cells experience a global reduction of transcription, e.g., during the DNA damage response [148]. This does not preclude execution of a specific transcriptional response but such incompletely processed transcripts could ensure a more rapid one. Interestingly, the transcripts with detained introns were found to be enriched in DNA damage response, DNA repair and cell cycle genes, among others [147]. Moreover, DNA damage could trigger changes in the splicing of the transcripts with the detained introns [147]. Thus, it is conceivable that ERH could play a role in the removal of the detained introns present in the DNA damage response and cell cycle transcripts. Of note, CHTOP can bind its own pre-mRNA to retain one of the introns, the lack of PRMT5 activity led to significant increase in the levels of detained introns, and PRMTs inversely regulated intron detention through arginine methylation in CHTOP and the Sm proteins [102,149,150].

ERH is also involved in the formation of the repressive heterochromatin in order to prevent the untimely expression of the meiotic genes and to protect *rDNA* integrity in *S. pombe*. In *C. elegans*, to protect the genome integrity in the germline, the piRNA pathway with which ERH is associated blocks the unwanted transcription from dangerous non-self-sequences, such as transposons, by the formation of the repressive heterochromatin. This is also the case in mammals, in which the formation of the repressive heterochromatin in the ERH-dependent manner serves to prevent the untimely expression of the lineage-specific genes, including meiotic and gametogenic ones, and to curb many repetitive elements. However, in *S. pombe* ERH is required for the assembly of the heterochromatin at some loci only (the heterochromatin islands at the meiotic genes), while the formation of most of the heterochromatin, especially the constitutive one and at retrotransposon loci, depends on a different mechanism that is based on the RNAi pathway with the RITS and RDRP complexes, the latter being absent from metazoans [151,152]. Thus, it seems that in humans the ERH function from *S. pombe* is extended to other lineage-specific genes, while the repression of the repetitive elements is the new one. Moreover, it is possible that ERH could also be involved in the formation of the heterochromatin mediated by chromatin-associated RNAs at transposon loci and during the inactivation of the whole chromosome X [130]. Regardless of the latter, ERH is required for transcriptional silencing in organisms ranging from the fission yeasts to humans.

Moreover, apart from affecting nuclear DNA replication through the regulation of expression levels and the splicing of some DNA metabolism genes, including DNA replication genes, ERH could also be involved in the assembly of the replication complex and/or in the DNA synthesis and its tethering to the nuclear matrix by its interactions with CIZ1, PDIP46/SKAR and SAFB1/2. However, the action(s) of ERH in the context of these proteins certainly needs more study. Since the activity of ERH seems to be associated with RNA in numerous cases, one can speculate that these processes could also be mediated by chromatin-associated RNAs. In fact, some ncRNAs, like Y RNAs, were implicated in the initiation of the nuclear DNA replication [153]. On the other hand, CIZ1 and SAFB1/2 are also involved in RNA metabolism by regulation of the ERα transcriptional activity, albeit in the opposite manner, and CIZ1 and SAFB1 participate in the functioning of Xist. However, even more significantly, PDIP46/SKAR, SAFB1, CIZ1 and RPS3 have been demonstrated, or at least suggested, to be required for responses to DNA damage and replication stress, and for DNA repair.

Lastly, the original mutation in the *e(r)* gene in *D. melanogaster* manifested only at pyrimidine deficiency, a condition with the dNTP imbalance/lowered dNTP pool that causes DNA replication stress, as noted elsewhere [50,55].

Taken together, the ERH interactome is a quite extensive network of protein–protein interactions that do not seem to be limited to mRNA splicing and mitosis, as is suggested by the current official ERH name. In fact, these interactions could ensure a much broader role of ERH that consists in the participation in the pathways maintaining genome integrity in a preventive and repair manner, namely by the formation of the repressive heterochromatin, the biogenesis of the transcripts of the subset of genes involved in the DNA damage response and the DNA metabolism, and maybe even by direct interactions with several proteins needed for replication stress and DNA damage responses and DNA repair (Figure 4). Therefore, it is proposed here that ERH, in addition to all its other known activities, should be considered as an important genome integrity protector protein.

## Data Availability

Not applicable.
